# Correction of Griscelli Syndrome Type 2 causing mutations in the *RAB27A* gene with CRISPR/Cas9

**DOI:** 10.55730/1300-0152.2705

**Published:** 2024-07-31

**Authors:** Özgür Doğuş EROL, Şimal ŞENOCAK, Burcu ÖZÇİMEN, Gülen GÜNEY ESKEN, Hasan Basri KILIÇ, Çetin KOCAEFE, Niek P. VAN TIL, Fatima AERTS KAYA

**Affiliations:** 1 Department of Stem Cell Sciences, Center for Stem Cell Research and Development, Graduate School of Health Sciences, Hacettepe University, Ankara, Turkiye; 2 Hacettepe University Advanced Technologies Application and Research Center, Hacettepe University, Ankara, Turkiye; 3 Inserm UMR-S-1310, Villejuif, France; 4 Department of Medical Biology, Faculty of Medicine, Hacettepe University, Ankara, Turkiye; 5 Amsterdam Leukodystrophy Center, Emma Children’s Hospital, Amsterdam University Medical Center, Amsterdam Neuroscience, Amsterdam, the Netherlands; 6 Department of Integrative Neurophysiology, Center for Neurogenomics and Cognitive Research, Vrije Universiteit Amsterdam, Amsterdam, the Netherlands

**Keywords:** CRISPR/Cas9, electroporation, homology-directed repair, nonhomologous end joining

## Abstract

**Background/aim:**

Griscelli Syndrome Type 2 (GS-2) is a rare, inherited immune deficiency caused by a mutation in the *RAB27A* gene. The current treatment consists of hematopoietic stem cell transplantation, but a lack of suitable donors warrants the development of alternative treatment strategies, including gene therapy. The development of mutation-specific clustered regularly interspaced palindromic repeats (CRISPR)/Cas9 gene editing technology has opened the way for custom-designed gene correction of patient-derived stem cells. In this study, we aimed to custom design CRISPR/Cas9 constructs and test their efficiency on homology-directed repair (HDR) on the correction of exon 3 and exon 7 mutations in the *RAB27A* gene of GS-2 patient-derived mesenchymal stem cells (MSCs) and induced pluripotent stem cells.

**Materials and methods:**

We assessed *RAB27A* gene and protein expression using qRT-PCR, Western Blot, and immune fluorescence in GS-2 patient-derived MSCs and induced pluripotent stem cells (iPSCs). Guide RNAs (gRNAs) and donor DNAs were designed based on patient mutations in exon 3 and exon 7 using the CHOPCHOP online tool and transfected into GS-2 MSCs and iPSCs by electroporation. The cells were cultured for 2 days and then used for mutation analysis using DNA sequencing.

**Results:**

MSCs and iPSCs from the GS-2 patients lacked *RAB27A* gene and protein expression. After gRNA and donor DNAs were designed and optimized, we found HDR efficiency with gRNA3.3 (10% efficiency) and gRNA7.3 (27% efficiency) for MSCs but lower efficiency in iPSCs (<5%). However, transfection of both MSCs and iPSCs resulted in massive cell death, loss of colony formation, and spontaneous differentiation.

**Conclusion:**

The use of CRISPR/Cas9 to genetically correct MSCs and iPSCs from GS-2 patients with different mutations through HDR is feasible but requires optimization of the procedure to reduce cell death and improve stem cell function before clinical application.

## 1. Introduction

In recent years, the development of a wide variety of gene therapeutic strategies for treating (rare) inherited diseases has advanced rapidly. Several gene therapeutic advanced therapy medicinal products for these diseases have now received marketing authorization from the FDA, including Roctavian and Hemgenix, for treating hemophilia A (fVIII deficiency) and B (fIX deficiency), respectively, Luxturna for retinal dystrophy, Skysona for juvenile cerebral adrenoleukodystrophy (CALD), Vyjuvec for epidermolysis bullosa, Zynteglo for ß-thalassemia, and Zolgensma for spinal muscular atrophy^1^. The viral vectors used in these gene therapy products are chosen depending on their cellular target and range from the nonintegrating adeno-associated viral vectors (Hemgenix, Luxturna, Roctavian, Zolgensma) and herpes-simplex virus type-1 vectors (Vyjuvec) to the integrating lentiviral vectors (Skysona, Zynteglo). In contrast to adeno-associated viral (AAV) and HSV-1-based vectors, used in vivo, lentiviral vectors are used in vitro to transduce autologous CD34+ hematopoietic stem cells (HSCs). However, it is very difficult to obtain sufficient (hematopoietic stem) cells to design viral vectors and test their transduction/protein expression efficiency for ultra-rare diseases. Therefore, the development of gene therapy for these types of rare diseases is currently lagging. The generation of induced pluripotent stem cells (iPSC) through ectopic overexpression of a combination of transcription factors, such as OCT4, SOX2, KLF4, and c-MYC (OSKM) (Takahashi et al., 2007), has allowed reprogramming and indefinite culture of somatic cells obtained from patients with ultra-rare diseases and the creation of indispensable biobanks as a source for these cells (Saito et al., 2023).

One of these ultra-rare diseases is an immune deficiency called Griscelli Syndrome Type 2 (GS-2). GS-2 is caused by a mutation in the *RAB27A* gene, which encodes a small Rab GTPase; this plays a vital role in exocytosis and intracellular membrane trafficking (Stinchcombe et al., 2001; Kimura and Niki, 2011). Although not all mutations in the *RAB27A* gene cause GS-2 (Al-Mofareh et al., 2020), several mutations have been linked to the development of immune dysfunction of neutrophils, macrophages, and cytotoxic T cells combined with hyperactivation of the immune system, resulting in hemophagocytic lymphohistiocytosis (Ménasché et al., 2000). Although mutations in the *RAB27A* gene may affect multiple cell types outside of the hematopoietic/immunologic system, including melanocytes (Haddad et al., 2001) and most secretory cells (Hume et al., 2001), the major clinical issues linked to this mutation are related to dysfunction of the immune system (Stinchcombe et al., 2001). Therefore, HSC transplantation is the treatment of choice for children diagnosed with GS-2. However, in the absence of a suitable, matched donor, reintroducing *RAB27A* expression into the stem cells of the hematopoietic system could potentially treat *RAB27A* deficiency. Since the introduction of *RAB27A* into HSCs requires permanent gene integration into the genome to obtain consistent *RAB27A* expression in the progeny of the transduced cells, gene therapy for GS-2 using viral vectors would ideally be done using lentiviral vector systems (Aiuti et al., 2013). Although lentiviral vector systems have been proven highly effective in the development of treatments for certain metabolic (e.g., CALD) and hematopoietic diseases (e.g., b-thalassemia/sickle cell disease), the potential risks related to lentiviral integrations sites, such as integrational mutagenesis (Hacein-Bey-Abina et al., 2003) or risks related to overexpression of the transgene (Brody and Waldman, 2006) have led researchers to develop other methods for direct, patient-specific, and mutation-specific repair, such as the novel clustered regularly interspaced palindromic repeats (CRISPR)/Cas9 gene editing technology (Sternberg et al., 2014).

The CRISPR/Cas9 system was initially described as an adaptive immune system in prokaryotes (Jinek et al., 2012), but its precise genome editing capacities have since been enhanced and developed into rapid and powerful gene editing tools (Gundry et al., 2016). Essentially, the CRISPR tool consists of a designer single-guide RNA (sgRNA) that consists of a tracrRNA and crRNA interacting with the Cas9 nuclease. When the sgRNA/Cas9 complex binds to its target DNA, the Cas9 enzyme cuts the genome, creating double-strand breaks with high efficiency and relatively low off-target effects (Hossain, 2021).

In this study, we aimed to assess the efficacy of CRISPR/Cas9 gene editing to repair the mutations of two GS-2 patients with mutations in the exon 3 and exon 7 of their *RAB27A* gene using GS-2 bone marrow mesenchymal stem cells (MSCs) and induced pluripotent stem cells (iPSCs) that we previously generated and characterized in another study (Güney-Esken et al., 2021) to reveal the feasibility of using CRISPR/Cas9 gene editing as a tool for mutation directed gene therapy of GS-2.

## 2. Materials and methods

### 2.1. Human control and GS-2 MSC isolation

Primary bone marrow MSCs from healthy donors and GS-2 patients were obtained following approval by Hacettepe University’s Ethical Committee for Non-Interventional Clinical Research (GO14/424) and informed consent (Güney-Esken et al., 2021). MSCs were cultured with DMF10, consisting of a mixture of 60% Dulbecco’s Modified Eagle Medium-Low Glucose (#31885, Thermo Fisher Scientific, Waltham, MA, USA), 40% MCDB-201 (#M6770, Sigma-Aldrich Corps., St. Louis, MO, USA) medium, supplemented with 10% heat-inactivated fetal bovine serum (#10270, FBS-HI, Life Technologies, Carlsbad, CA, USA), 1% penicillin/streptomycin (#15140, P/S, Gibco, Grand Island, NY, USA) and 2 mM L-glutamine (#G3126, Sigma-Aldrich Corps, St. Louis, MO, USA). Media were changed twice a week. When the cells reached 70%–80% confluency, they were passaged using 0.25% Trypsin/1 mM EDTA. Adipogenic and osteogenic differentiation of the MSCs was initiated, as described in a previous study (Güney-Esken et al., 2021).

### 2.2. Human control and GS-2 iPSC culture

The control and GS-2 patient-derived iPSCs used in this study were generated using the transfer of OSKM with lentiviral vectors, previously characterized in detail (Güney-Esken et al., 2021). Mutation analysis confirmed the ongoing presence of disease-related mutations in the *RAB27A* gene after iPSC generation. The iPSC clones used in this study were YF/#A2C3 (mutation: c.148-149delAGinsC in exon 3) and IK/clone #5 (mutation: c.514_518delCAAGC in exon 7). The reuse of these cells was approved by Hacettepe University’s Ethical Committee for Non-Interventional Clinical Research (GO20/316). In brief, iPSCs were cultured on Matrigel-coated (#354277, Corning Inc., NY, USA) 6-well plates in TeSR-E8 medium (#05991, STEMCELL Technologies, Vancouver, Canada) and supplement (#05990, STEMCELL Technologies, Vancouver, Canada). GS-2 iPSCs were passaged as aggregates using ReLeSR cell dissociation solution (100-0484, STEMCELL Technologies, Vancouver, Canada), according to the manufacturer’s instructions. The collected cell aggregates were then maintained at 37 °C with 5% CO_2_, and media were changed daily. Before transfection, iPSCs were plated onto Matrigel-coated 6-well plates and cultured in TeSR-E8 Plus media containing 10-μM ROCK inhibitor (#1254, Tocris Bioscience, Bristol, UK).

### 2.3. qRT-PCR

MSCs from healthy donors and GS-2 patients were assessed for *RAB27A* gene expression only; iPSCs from healthy donors and GS-2 patients were assessed for *RAB27A* expression, as well as for expression of the *OCT4, SOX2*, and *NANOG* pluripotency genes. Briefly, RNA isolation was performed with a Direct-Zol RNA isolation kit (#R2062, Zymo Research, Irvine, CA, USA) according to the manufacturer’s protocol. RNA was reverse transcribed to cDNA, and qRT-PCR was performed using the GoTaq 2-Step RT-qPCR kit (#A6010, Promega Corps., Madison, WI, USA) and LightCycler 480 Probes Master mix (#04707494001, Roche, Basel, Switzerland). Samples were assessed using a Light Cycler 480 II (Roche, Basel, Switzerland). The primer sequences used are shown in Table S1. *GAPDH* and/or *B2M* were used as a housekeeping gene for normalization, and the 2^−ΔΔCT^ method was used to calculate the relative gene expression. A Student’s t-test analysis was performed to determine statistical significance (p-value < 0.05) for differences between the two groups. qRT-PCR analysis was initiated, as described above. All calculations were performed using the Microsoft Excel (Microsoft, Redmond, WA, USA) spreadsheet program.

### 2.4. gRNA design and T7 endonuclease assay

The gRNA sequences targeting the *RAB27A* gene exon 3 and exon 7 mutations were designed using the CHOPCHOP website (https://chopchop.cbu.uib.no) (Labun et al., 2019). The GS-2 mutations were annotated on the *RAB27A* DNA coding sequence using SnapGene software (GSL Biotech, San Diego, CA, USA). For each mutation, three mutation-specific gRNAs were designed and tested on the GS-2 MSCs. Transfections were performed using the Neon Transfection System Kit (#MPK10096, Thermo Fisher Scientific, Waltham, MA, USA). Briefly, gRNAs were diluted in IDTE buffer in the presence of 200-mM crRNA and 200-mM tracrRNA to form the single-guide RNA (sgRNA) complex. The sgRNA complexes were then incubated with Cas9 enzyme for 15 min at room temperature to allow the formation of the ribonucleoprotein (RNP) complex. After transfection, the cells were incubated at 37 °C with 5% CO_2_ for 72 h. Cells from a single well were pooled and used to extract genomic DNA (gDNA). In brief, samples were incubated overnight with nuclear lysis buffer (50 mM Tris-Cl, 10 mM EDTA, 0.8% SDS) and then digested with phenol/chloroform/isoamyl alcohol (25:24:1) for 2 min. After centrifugation, the samples were transferred to 100% ethanol to precipitate the DNA. gDNA was used for PCR and loaded onto agarose gels. When PCR bands were visible on the gel, we performed the T7 Endonuclease I (T7E1) assay (#M0302, New England Biolabs, Ipswich, MA, USA) to assess the genome targeting efficiency of the designed gRNAs. Amplicons were tested using gDNA from the target GS-2 MSCs and negative control healthy donor MSC DNA. After the assay, nonhomologous end joining (NHEJ) events were calculated from the agarose gel images using Gel Analyzer software (http://www.gelanalyzer.com/), followed by mismatch detection with the T7EI calculator (https://horizondiscovery.com/en/ordering-and-calculation-tools/t7ei-calculator). The estimated gene modification rate was calculated using the following formula: % gene modification = 100 × (1-(1-fraction cleaved)) (Guschin et al., 2010). Approximately 110 nt ssODN repair templates were designed with homologous genomic sequences obtained from NCBI (Gene ID: 5873) for selected gRNAs.

### 2.5. Correction of *RAB27A* mutations using CRISPR/Cas9

For correction of GS-2 MSCs, cells were collected with Trypsin/EDTA, and single cells were resuspended in R-solution. For correction of GS-2 iPSCs, iPSCs from a single well were collected with ReLeSR solution, and cell aggregates were resuspended in R-solution. The RNP complex was prepared from a mixture of gRNA (50 mM), Cas9 (61 mM), and PBS. The cell suspension was mixed with the RNP complex, homology-directed repair (HDR) donor DNA at a concentration of 100 mM with an electroporation enhancer (#1075916, Integrated DNA Technologies, Coralville, IA, USA ) at 20 mM and pipetted onto the Neon Transfection System. For negative controls, DMSO was used instead of donor DNA. Transfections were performed at 1400 V, 10 ms, and three pulses. Transfected MSCs were cultured in 6-well plates in DMF10 without penicillin/streptomycin for 2 days. After transfection of the iPSCs, the cells were placed on fresh Matrigel-coated dishes and cultured in TeSR-E8 with 10-mM ROCK inhibitor for 2 days.

### 2.6. Mutation repair analysis using Sanger and next-generation sequencing

After 2 days of culture, transfected and control GS-2 MSCs and iPSCs were collected, and gDNA was isolated using phenol: chloroform: isoamyl alcohol (25: 24: 1) extraction, as described above. Sequencing was done using gDNA via Sanger and MiSeq (Illumina, San Diego, CA, USA). Aligned “.bam” files were analyzed with IGV 2.3 (Broad Institute, Cambridge, MA, USA) software.

## 3. Results

### 3.1. Assessment of *RAB27A* expression by GS-2 MSCs and iPSCs

The healthy donor and the GS-2 MSCs and iPSCs used in this study were previously characterized in detail (Güney-Esken et al., 2021). We did not observe any specific changes in cell proliferation or morphology of the GS-2 MSCs or iPSCs compared to healthy donor MSCs and iPSCs (Figure 1). However, since the *RAB27A* protein and gene expression levels in these cells were not previously assessed, we measured the baseline protein expression levels of *RAB27A* in healthy donor and GS-2 MSCs (Figures S1 and S2) and the gene expression levels of *RAB27A* in healthy donor and GS-2 MSCs and iPSCs (Figure 2). *RAB27A* protein expression in healthy donor MSCs was found to be relatively low in comparison to cancer cell lines, such as K562; however, in comparison to the healthy control, MSCs expression was considerably decreased in the MSCs samples of the two GS-2 patients (İK and YF).

We previously demonstrated that GS-2 patient-derived iPSC clones represent the GS-2 genotype faithfully since mutation analysis confirmed the presence of *RAB27A* mutations in these cell lines (Güney-Esken et al., 2021). Accordingly, we found a high expression of the *OCT4*, *SOX2*, and *NANOG* pluripotency genes in the GS-2 iPSCs but decreased expression of *RAB27A* in both GS-2 patient-derived iPSC clones in comparison to the healthy iPSC control sample (Figure 2).

### 3.2. gRNA and donor DNA design and assessment of genome targeting efficiency

For each patient (exon 3; c.148-149delAGinsC and exon 7; c.514-518delCAAGC), we designed three different mutation-specific gRNAs (Table 1). We used the CHOPCHOP online tool to design all gRNAs using annotation of the GS-2 mutations on the *RAB27A* DNA coding sequence (Figure S3). We then tested the genome targeting efficiency of the three selected gRNAs for each exon using the T7 endonuclease assay. Using gel analyzer software, we found the highest efficiency with gRNA 3.3 (10% efficiency) and gRNA 7.3 (27% efficiency) (Figure 3). Based on these data, we used the HDR donor DNA sequences, as shown in Table 2.

### 3.3. Transfection of the RNP complex and donor DNA into GS-2 MSCs and iPSCs

We then proceeded to test the efficiency of the HDR on GS-2 patient-derived MSCs using the designed RNP complexes or DMSO controls. Cells were allowed to recover for 2 days after transfection and then used for DNA mutation analysis. Despite the positive results with the gRNAs designed to correct exon 3 MSCs in the T7 endonuclease assay, we determined that no correction of exon 3 existed following HDR (Figure 4A). Although we found HDR of some of the cells with mutations in exon 7 MSCs, we also observed that a considerable fraction of the cells had obtained deletions in the gene (Figure 4B). After optimizing the procedures with different donor DNA concentrations, we decided to test HDR efficacy on the GS-2 iPSCs. After transfection, the exon 3 mutant iPSCs were cultured for 2 days, but, despite having added ROCK inhibitor, we observed massive iPSC cell death, decreased colony formation, and spontaneous differentiation (Figure 5, part A). Sequencing analysis revealed the presence of HDR in a minority of the transfected cells (Figure 5, part B).

## 4. Discussion

The absence of sufficient cellular research material has hampered the development of gene therapy for ultra-rare genetically inherited diseases. However, the critical discovery of cellular reprogramming and the creation of iPSCs have largely replaced the need for primary patient material (Takahashi et al., 2007). Using bone marrow-derived MSCs, we previously created several GS-2 iPSC lines from two patients with different mutations in exon 3 and exon 7 of their *RAB27A* gene, respectively (Güney-Esken et al., 2021). The current study explored the possibility of using novel CRISPR/Cas9-mediated gene correction technology (Jinek et al., 2012) on both GS-2 MSCs and iPSCs. We designed and tested three different gRNA constructs for each exon (i.e. exon 3 and exon 7) and tested the gene correction efficacy through HDR.

CRISPR/Cas9 technology has made it possible to both knock-out or knock-in a specific sequence of nucleotides. CRISPR/Cas9 has proved highly efficient as a knock-out system, using NHEJ as its primary DNA repair mechanism. However, repair of genetic mutations using a site-directed knock-in is more difficult to develop. Many researchers have struggled due to off-target effects and encountered low HDR efficiency (Smith et al., 2015), especially in patient-derived primary (stem) cells (Xu et al., 2018). In contrast, genome editing efficiency has been achieved with higher success rates when using tumor-derived cell lines, such as 293T cells or K562, than with iPSC or embryonic stem cell (ESC) lines (Hsu et al., 2013; Mali et al., 2013). Studies attempting to improve gene transfer methods for iPSC/ESCs, including electroporation/nucleofection, lipofection, or the use of magnetic or nanoparticles, have led to inconsistent results and are likely more affected by the type of construct used than the gene transfer method itself (Villa-Diaz et al., 2010).

In this study, we found that although gene correction using viral-free methods to transfer the RNP complex and the donor DNA to GS-2 MSCs and iPSCs is feasible, with correction rates exceeding those previously published for iPSCs (Hsu et al., 2013; Mali et al., 2013), the technique is harmful to MSCs and iPSCs since the latter displayed a rapid decrease in cellular viability, despite the presence of optimal cell culture media and the addition of ROCK inhibitor to prevent cell dissociation-induced apoptosis (Watanabe et al., 2007). In addition to cell death, the iPSCs showed uncontrolled differentiation after nucleofection (Li et al., 2018). Although the efficiency rates of HDR in our settings were acceptable, sufficient room for improvement exists for this procedure. Since the HDR rate depends on cell cycling, with most HDR activation limited to the S/G2 stages (Saleh-Gohari and Helleday, 2004), actively proliferating cells, including stem cells, is preferred (Benati et al., 2022). Furthermore, methods inducing cell cycle synchronization or arrest cells in the S/G2 phase, as well as introducing specific selection markers or adding NHEJ inhibitors, may increase the relative contribution of HDR (Liu et al., 2018; Di Stazio et al., 2021; Chen et al., 2022). Other factors that may improve the HDR rate and efficiency include the use of single-stranded oligo DNA nucleotides and specially designed donor DNA with asymmetric homologous arms, such as the constructs used in this study (Richardson et al., 2016). However, even if HDR rates in iPSCs or ESCs are increased, cells may still have to recover from the effects of electroporation with editing plasmids and often respond with induction of massive apoptosis (Li et al., 2018). Therefore, using viral vectors, such as AAV vectors, to overcome this problem has been suggested (Bak et al., 2017).

This study shows that although it is possible to custom design CRISPR/Cas9 constructs to precisely fit the mutation and correct the gene of interest, improvement of HDR efficiency and methods to enhance cell survival after gene correction require further attention to make the procedure clinically feasible. Thus, although the current techniques are beneficial for knocking out a gene, modeling a disease, or creating a mutation, current methods for site-directed knock-in with sufficient efficiency and high-cell viability still need to be optimized. In addition, since patients with rare genetic diseases—with their unique mutations—require a personalized design of the constructs, as well as testing on and selection of patient-derived cells, the use of the current CRISPR/Cas9 technology for treating ultra-rare genetic disorders may be too laborious and not cost-effective. However, the use of CRISPR/Cas9 to cure or alleviate the symptoms of a disease by disrupting another gene, as is the case with the first approved CRISPR treatment, Casgevy, developed for treating patients with sickle-cell disease or β-thalassemia, may be more widely applicable (Frangoul et al., 2021).

In conclusion, developing custom-designed CRISPR/Cas9 gene therapy for treating ultra-rare diseases, such as GS-2, is feasible, but improving HDR efficiency and cell viability after transfection requires further optimization.

## Supplementary Data

**Table S1:** Forward and reverse sequences of genomic PCR and qRT-PCR primers.

Gene	Forward sequence (5’à3’)	Reverse sequence (5’à3’)	Amplicon size
*RAB27A Exon 3 (genomic)*	GAGACTCTGGTGTAGGGAAGA	GATCCCAACCTTTGTCCTCCT	209
*RAB27A Exon 7 (genomic)*	ATCAAGAGCAAAGGTCACTCTC	GGGCCACCTGAACTACTATG	301
*GAPDH*	CATCACTGCCACCCAGAAGAC	TGACCTTGCCCACAGCCTTG	122
*RAB27A*	CTGAAGAGGACATGTGATTGGA	GTCTTTGAGCCTTAGATTTCCAG	136
*B2M*	CCGTGTGAACCATGTGACTTT	CCTCCATGATGCTGCTTACA	Probe primer
*POU5F1*	GCAAAACCCGGAGGAGTC	TCCCAGGGTGATCCTCTTCT	Probe primer
*SOX2*	ATGGGTTCGGTGGTCAAGT	GGAGGAAGAGGTAACCACAGG	Probe primer
*NANOG*	ATGCCTCACACGGAGACTGT	CTGCAGAAGTGGGTTGTTTG	Probe primer

Figure S1 Donor and GS-2 MSCs *RAB27A* protein expression. To confirm the absence of *RAB27A* protein expression in the GS-2 MSCs and determine baseline levels of *RAB27A* protein expression in the healthy donor MSCs, we performed Western Blots. As positive controls, we used the *RAB27A* expressing cell line K562. In brief, we collected donor MSCs (donor 1 and donor 2), GS-2 MSCs (YF ad İK), K652 cells in RIPA buffer (#89900, Thermo Fisher Scientific, Waltham, MA, USA), and the measured protein content with a BCA protein assay kit (#23227, Thermo Fisher Scientific, Waltham, MA, USA) using a Nanodrop 1000. Denaturized proteins were separated using SDS-PAGE and transferred onto PVDF membranes using the Trans-blot Turbo Blotting system (Bio-Rad, Hercules, CA, USA). Membranes were stained with an anti-*RAB27A* antibody (#STJ25258, St John’s Laboratory Ltd, London, UK) at 1:1000 overnight at 4 °C and counterstained with secondary antirabbit-HRP antibody at 1:10000 (#11-4220-82, Invitrogen, Waltham, MA, USA) for 1 h at room temperature. Peroxidase activity was measured using the Clarity Western ECL Substrate kit (#32132, Thermo Scientific, Waltham, MA, USA). Blot 1 was stained consecutively with GAPDH and *RAB27A*, respectively. Blot 2 was stained simultaneously with both GAPDH and *RAB27A*. In addition, this blot contained data used for another study. Therefore, in the figure showing combined data from Blot 1 and Blot 2, we removed these data. The graph representing relative protein expression compared to GAPDH was calculated for each sample using the ImageJ program (NIH, Java, 2022).

Figure S2 Immunofluorescence staining of GAPDH and *RAB27A* in donor and GS-2 MSCs. To detect *RAB27A* protein expression in healthy donor and GS-2 MSCs, the cells were seeded at 15,000 cells/well in 8-well chamber slides. The cells were fixed with 4% paraformaldehyde (#8187085000, Sigma-Aldrich Corps, St. Louis, MO, USA) and permeabilized with 0.1% Triton X-100 (CAS # 9036-19-5, Merck, Rahway, NJ, USA) in PBS. Cells were stained with anti-GAPDH (#MA5-15738, Invitrogen, Waltham, MA, USA) and anti-*RAB27A* (#25258, St John’s Laboratory Ltd, London, UK) primary antibodies at 1:100. The secondary antibodies goat antirabbit IgG (#ab175471) and goat antimouse IgG (#ab175473) were diluted at 1:1000. Nuclei were counterstained with DAPI (#D8417-5MG, Sigma-Aldrich Corps, St. Louis, MO, USA) at 5 mg/mL (blue color). Photographs were taken with an inverted microscope (Olympus LS, IX73, Olympus, Shinjuku City, Japan) and analyzed using ImageJ software (NIH, Java, 2022). Upper panel: healthy donor MSCs showing bright expression of GAPDH and *RAB27A*; middle panel: GS-2 MSCs (YF) showing bright staining of GAPDH but an absence of *RAB27A* staining; lower panel: fluorescent photographs of donor and GS-2 MSCs (YF) after staining with primary anti-*RAB27A* antibody only (no signal), secondary antibody only (no signal), and both (positive for donor MSCs and negative for GS-2 MSCs), indicating the use of appropriate laboratory staining procedures.

Figure S3 gRNA design using the CHOPCHOP online tool. To design gRNAs, the coding sequence of *RAB27A* was annotated with patient mutations and uploaded to CHOPCHOP. This tool provides the gRNA sequences according to their efficiency (the likelihood of cutting facilities) and specificity (the likelihood of off-target sites). Based on these data, we selected the best-ranking sequences near or on the targeted mutation sites for *RAB27A* exon 3 (left) and *RAB27A* exon 7 (right). Prediction of probable efficiency and mismatch (MM) scores for the constructs designed to target exon 3 (upper panel) and exon 7 (lower panel) mutations are shown in the lower panel. Green boxes represent the sequences near the annotated mutation site.

## Figures and Tables

**Figure 1:**
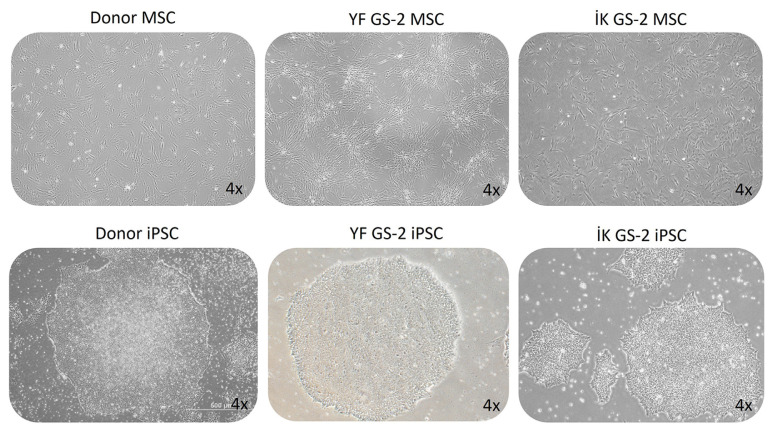
Morphology of healthy donor and GS-2 patient-derived MSCs and iPSCs taken with a light microscope of donor and GS-2 MSC cultures (upper lane) and their iPSCs (lower lane). MSCs show typical fibroblast shapes, whereas iPSCs grow in distinct colonies, as shown in light microscope pictures from representative cultures.

**Figure 2:**
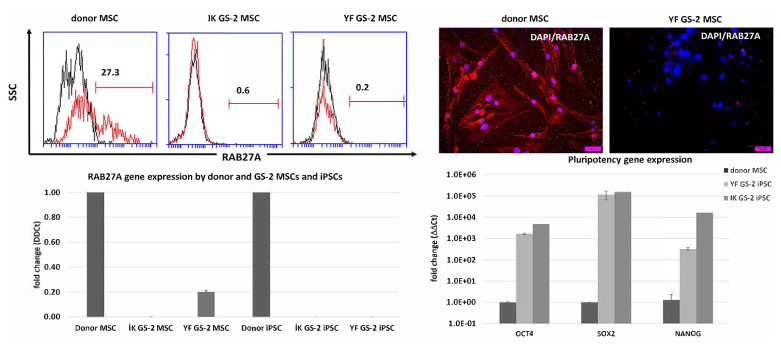
*RAB27A* expression by donor and GS-2 MSCs and iPSCs. Donor and GS-2 patient-derived MSCs (IK and YF) were stained for *RAB27A* expression using flowcytometry (upper left) and immunofluorescent staining of adherent cells in culture (upper right, DAPI: blue, *RAB27A*: red). *RAB27A* gene expression in GS-2 MSCs from two donors (İK and YF) was calculated relative to *RAB27A* expression in healthy donor MSCs, whereas GS-2 iPSCs expression of *RAB27A* was calculated relative to healthy donor iPSCs (lower left). GS-2 iPSC expression of pluripotency genes was confirmed for *OCT4, SOX2*, and *NANOG* (lower right).

**Figure 3:**
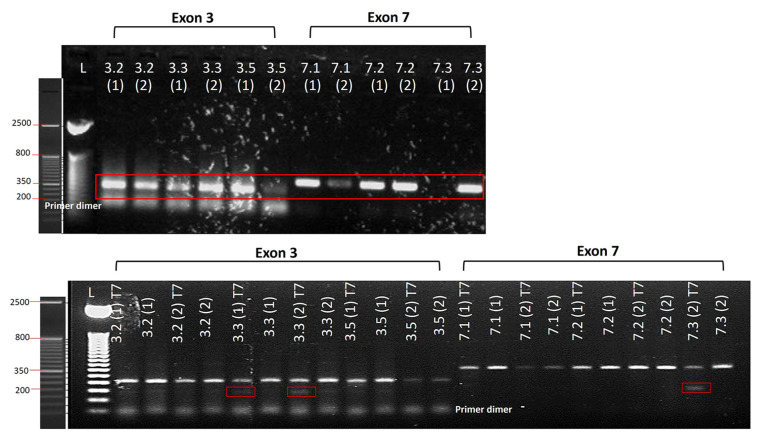
Genome targeting efficiency of different gRNAs. The genome targeting efficiency of the designed gRNAs (3.2, 3.3, and 3.5 for exon 3 and 7.1, 7.2, and 7.3 for exon 7) was tested using the T7 Endonuclease assay. Upper panel: the DNA of gRNA-transfected cells was controlled with PCR; lower panel: the PCR products were loaded onto a 2% agarose gel to detect gRNA efficiency after adding T7 endonuclease. All tests were run in duplicate. Genome targeting efficiency was the highest with gRNA 3.3 and 7.3 (as shown by the red rectangles in the lower panel).,

**Figure 4:**
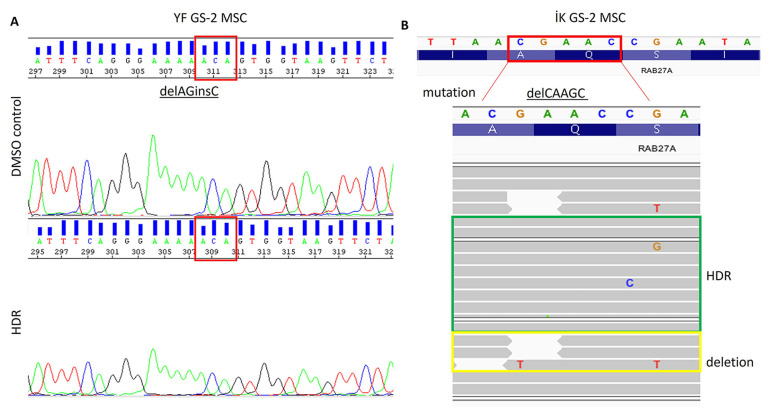
Mutation analysis of GS-2 MSCs after transfection. MSCs from two different GS-2 patients (YF: exon 3 delAGinsC; İK: exon 7 delCAAGC) were transfected and cultured for 2 days. Mixed cell populations from a single well were collected for DNA analysis. Sequencing analysis revealed the absence of HDR in exon 3 (A) but the presence of HDR in up to 50% of the cells with exon 7 mutations (B).

**Figure 5:**
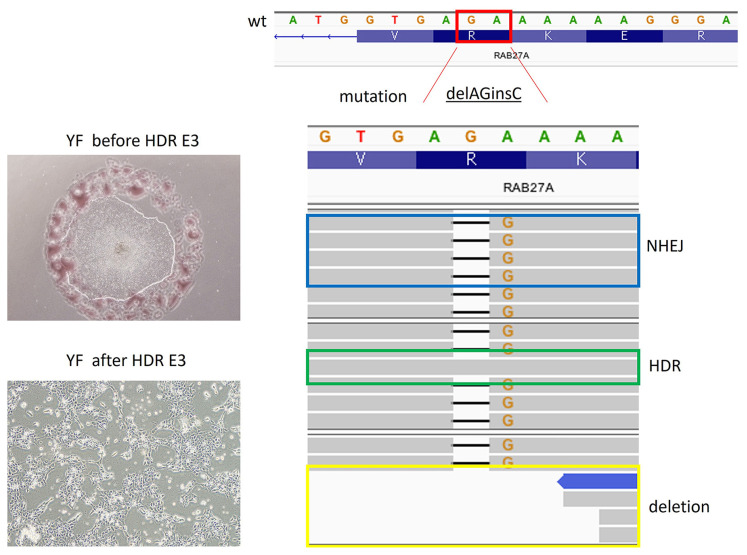
Transfection of GS-2 iPSCs with the *RAB27A* exon 3 mutation (upper left) resulted in loss of viability and spontaneous differentiation (lower left) and low HDR efficacy (right).

**Table 1:** gRNA sequences designed to target mutations in *RAB27A* exon 3 and 7.

Mutation site	gRNAs	crRNA sequences	PAM
Exon 3	gRNA3.2	TTGATTTCAGGGAAAAACAG	TGG
Exon 3	gRNA3.3	CTCCAAATTTATCACAACAG	TGG
Exon 3	gRNA3.4	CGATTACATTTTTACATAGA	AGG
Exon 7	gRNA7.1	ATAAGCAATTGAGATGCTTC	TGG
Exon 7	gRNA7.2	ATTGCTTATGTTTGTCCCAT	TGG
Exon 7	gRNA7.3	GGACCTGATAATGAAGCGAA	TGG

**Table 2:** Donor DNA sequences for HDR.

gRNA	Strand	Sequence
gRNA3.3	+	CAATATACAGATGGTAAATTTAACTCCAAATTTATCACAACAGTGGGCATTGATTTCAGGGAAAAAAGAGTGGTAAGTTCTATATCCTTCTATGTAAAAATGTAATCG
	−	CGATTACATTTTTACATAGAAGGATATAGAACTTACCACTCTTTTTTCCCTGAAATCAATGCCCACTGTTGTGATAAATTTGGAGTTAAATTTACCATCTGTATATTG
gRNA7.3	+	CTACTTTGAAACTAGTGCTGCCAATGGGACAAACATAAGCCAAGCAATTGAGATGCTTCTGGACCTGATAATGAAGCGAATGGAACGGTGTGTGGACAAGTCCTGGATTCCTGAAGG
	−	CCTTCAGGAATCCAGGACTTGTCCACACACCGTTCCATTCGCTTCATTATCAGGTCCAGAAGCATCTCAATTGCTTGGCTTATGTTTGTCCCATTGGCAGCACTAGTTTCAAAGTAG

## Footnotes

1 (Aerts-Kaya and van Til, 2023) (FDA (2023) Approved cellular and gene therapy products [online] website: https://www.fda.gov/vaccines-blood-biologics/cellular-gene-therapy-products/approved-cellular-and-gene-therapy-products [accessed 30-06-2023].
