# Supplementary figures and images for: Correction of Griscelli Syndrome Type 2 causing mutations in the RAB27A gene with CRISPR/Cas9
Source: Turk J Biol. 2024 Jul 31;48(5):290–8. doi: 10.55730/1300-0152.2705 (PMC11518329; doi:10.55730/1300-0152.2705)

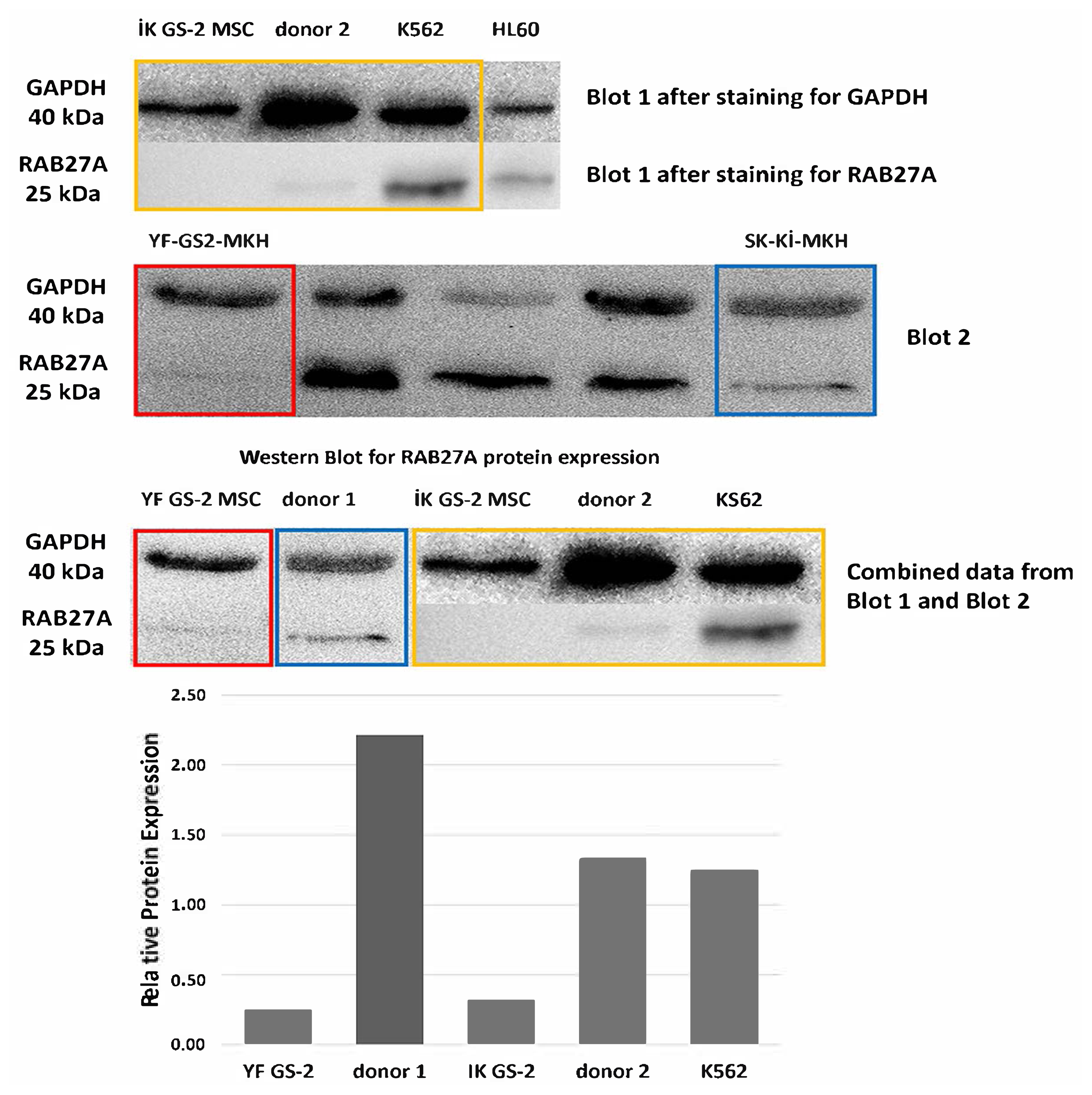

Supplement: Figure S1 — Donor and GS-2 MSCs RAB27A protein expression. To confirm the absence of RAB27A protein expression in the GS-2 MSCs and determine baseline levels of RAB27A protein expression in the healthy donor MSCs, we performed Western Blots. As positive controls, we used the RAB27A expressing cell line K562. In brief, we collected donor MSCs (donor 1 and donor 2), GS-2 MSCs (YF ad İK), K652 cells in RIPA buffer (#89900, Thermo Fisher Scientific, Waltham, MA, USA), and the measured protein content with a BCA protein assay kit (#23227, Thermo Fisher Scientific, Waltham, MA, USA) using a Nanodrop 1000. Denaturized proteins were separated using SDS-PAGE and transferred onto PVDF membranes using the Trans-blot Turbo Blotting system (Bio-Rad, Hercules, CA, USA). Membranes were stained with an anti-RAB27A antibody (#STJ25258, St John’s Laboratory Ltd, London, UK) at 1:1000 overnight at 4 °C and counterstained with secondary antirabbit-HRP antibody at 1:10000 (#11-4220-82, Invitrogen, Waltham, MA, USA) for 1 h at room temperature. Peroxidase activity was measured using the Clarity Western ECL Substrate kit (#32132, Thermo Scientific, Waltham, MA, USA). Blot 1 was stained consecutively with GAPDH and RAB27A, respectively. Blot 2 was stained simultaneously with both GAPDH and RAB27A. In addition, this blot contained data used for another study. Therefore, in the figure showing combined data from Blot 1 and Blot 2, we removed these data. The graph representing relative protein expression compared to GAPDH was calculated for each sample using the ImageJ program (NIH, Java, 2022). [file tjb-48-05-290s1.tif]

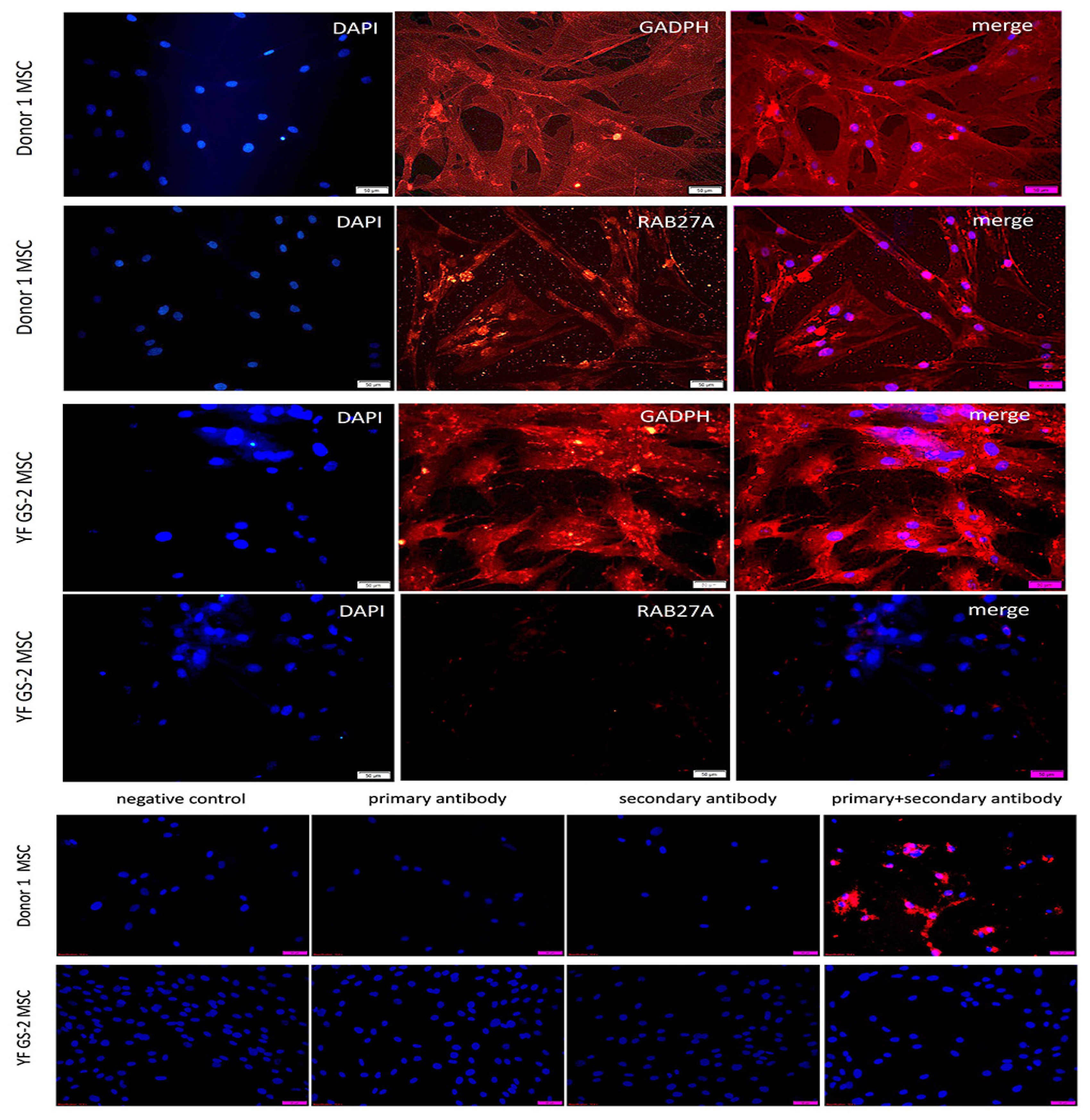

Supplement: Figure S2 — Immunofluorescence staining of GAPDH and RAB27A in donor and GS-2 MSCs. To detect RAB27A protein expression in healthy donor and GS-2 MSCs, the cells were seeded at 15,000 cells/well in 8-well chamber slides. The cells were fixed with 4% paraformaldehyde (#8187085000, Sigma-Aldrich Corps, St. Louis, MO, USA) and permeabilized with 0.1% Triton X-100 (CAS # 9036-19-5, Merck, Rahway, NJ, USA) in PBS. Cells were stained with anti-GAPDH (#MA5-15738, Invitrogen, Waltham, MA, USA) and anti-RAB27A (#25258, St John’s Laboratory Ltd, London, UK) primary antibodies at 1:100. The secondary antibodies goat antirabbit IgG (#ab175471) and goat antimouse IgG (#ab175473) were diluted at 1:1000. Nuclei were counterstained with DAPI (#D8417-5MG, Sigma-Aldrich Corps, St. Louis, MO, USA) at 5 mg/mL (blue color). Photographs were taken with an inverted microscope (Olympus LS, IX73, Olympus, Shinjuku City, Japan) and analyzed using ImageJ software (NIH, Java, 2022). Upper panel: healthy donor MSCs showing bright expression of GAPDH and RAB27A; middle panel: GS-2 MSCs (YF) showing bright staining of GAPDH but an absence of RAB27A staining; lower panel: fluorescent photographs of donor and GS-2 MSCs (YF) after staining with primary anti-RAB27A antibody only (no signal), secondary antibody only (no signal), and both (positive for donor MSCs and negative for GS-2 MSCs), indicating the use of appropriate laboratory staining procedures. [file tjb-48-05-290s2.tif]

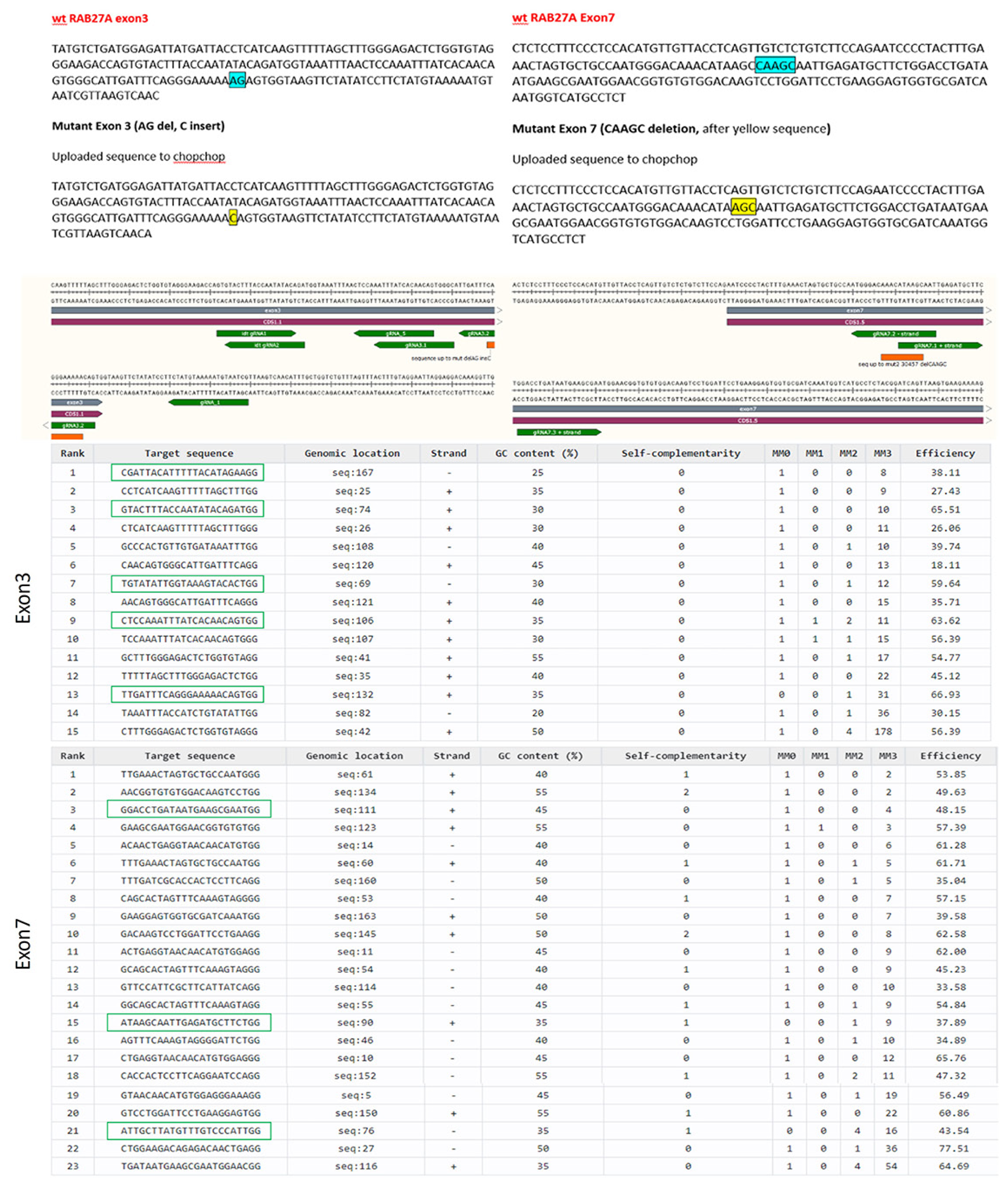

Supplement: Figure S3 — gRNA design using the CHOPCHOP online tool. To design gRNAs, the coding sequence of RAB27A was annotated with patient mutations and uploaded to CHOPCHOP. This tool provides the gRNA sequences according to their efficiency (the likelihood of cutting facilities) and specificity (the likelihood of off-target sites). Based on these data, we selected the best-ranking sequences near or on the targeted mutation sites for RAB27A exon 3 (left) and RAB27A exon 7 (right). Prediction of probable efficiency and mismatch (MM) scores for the constructs designed to target exon 3 (upper panel) and exon 7 (lower panel) mutations are shown in the lower panel. Green boxes represent the sequences near the annotated mutation site. [file tjb-48-05-290s3.tif]
